# Enhancing bioactivity of *Chlorella vulgaris* through enzymatic pretreatment and lactic acid fermentation

**DOI:** 10.1186/s40643-026-01050-3

**Published:** 2026-04-06

**Authors:** Hakki Bilgin, Shahana Aboobacker, Aušra Šipailienė, Vaida Kitrytė-Syrpa, Michail Syrpas

**Affiliations:** Department of Food Science and Technology, Kaunas University of Technology, Radvilenu pl. 19, Kaunas, Lithuania

**Keywords:** Microalgae, *Chlorella vulgaris*, Fermentation, Lactic acid bacteria, Enzyme pretreatment, Antioxidant capacity

## Abstract

This study explores an integrated bioprocessing approach to enhance the bioactivity of *Chlorella vulgaris* biomass through enzymatic pretreatment and lactic acid fermentation. Three pretreatment strategies—ultrasound-assisted hydrolysis, hydrothermal treatment, and enzymatic hydrolysis using Viscozyme, Alcalase, or their combination- were evaluated for their ability to release fermentable sugars and proteins. Enzymatic hydrolysis proved most effective, yielding up to 151.18 mg g DW^− 1^ of reducing sugars and 14.4 mg g DW^− 1^ of protein. Subsequent fermentation with *Lactiplantibacillus plantarum* and *Levilactobacillus brevis* demonstrated robust microbial growth (Δ log CFU ≈ 4.0) and significant acidification (pH 3.6–4.2), accompanied by lactic acid production up to 17.35 g/L. Functional characterisation revealed that combined enzymatic pretreatment and fermentation improved antioxidant properties, reaching values of 8.64, 19.46, 1.45 mg Trolox g DW⁻¹ in the CUPRAC, ABTS, and DPPH assays, respectively, while TPC reached 9.67 GAE g DW⁻¹. Moreover, Viscozyme-pretreated, *L. plantarum*-fermented samples showed inhibitory zones under non-neutralised screening conditions against Gram-positive bacteria. These findings highlight the combined effect of enzymatic pretreatment and microbial fermentation in enhancing the biofunctional value of *C. vulgaris*, offering a sustainable strategy for developing natural antioxidants and antimicrobial agents for food, nutraceutical, and cosmetic applications.

**Graphical Abstract:**

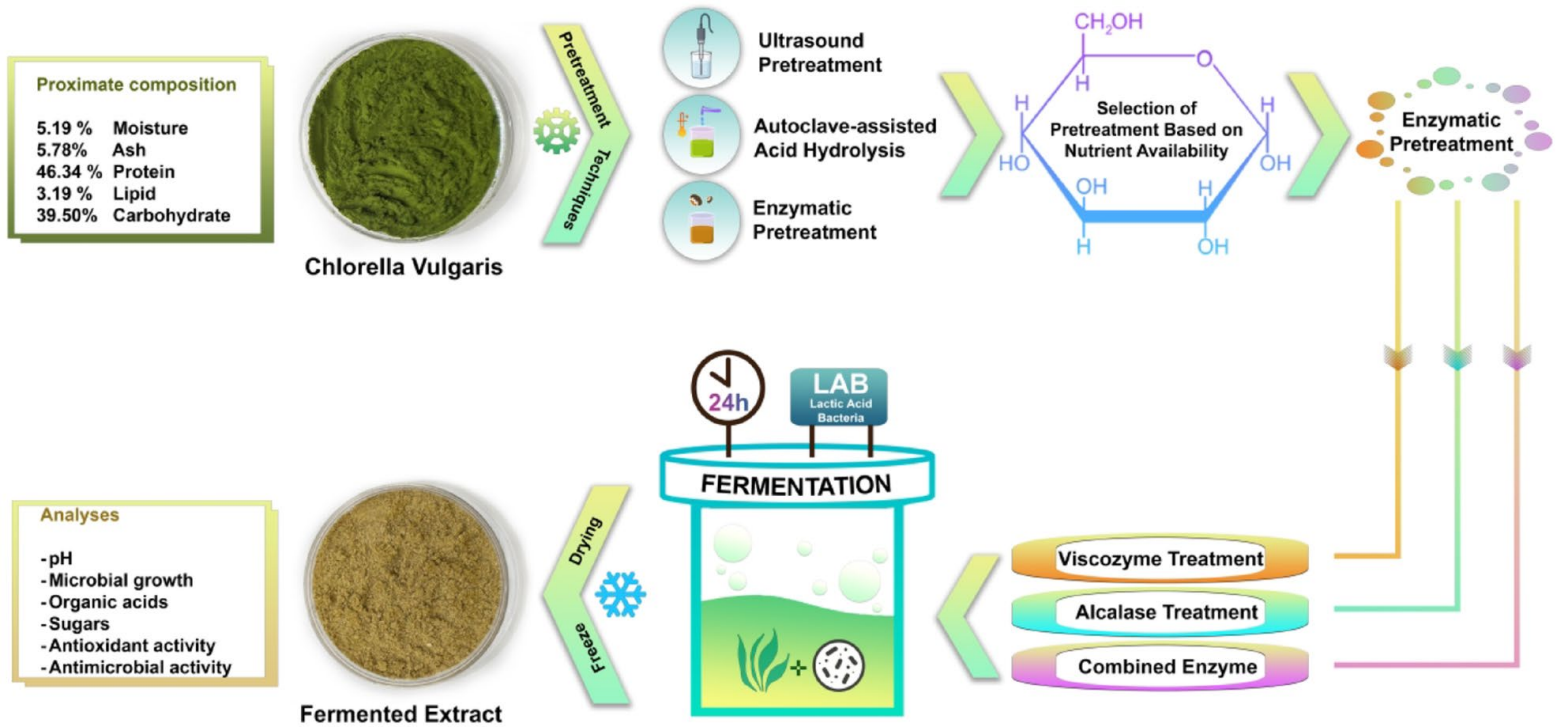

**Supplementary Information:**

The online version contains supplementary material available at 10.1186/s40643-026-01050-3.

## Introduction

In recent years, with the growing demand for sustainable, health-promoting food, there has been increased interest in microalgae as a renewable resource for food production. Beyond sustainability, microalgae, due to their nutrient-dense content and reported biofunctional properties, offer tremendous potential for applications in food, nutraceuticals, cosmetics, and pharmaceuticals (Sivaramakrishnan et al. 2025). Among microalgal species, *Chlorella vulgaris* is particularly attractive due to its high protein (≈ 43–58% DW) and carbohydrate (≈ 12–55% DW) content, as well as its role as a source of functional lipids, water-soluble vitamins and pigments (Wang et al. 2024). Its carbohydrate profile is rich in starch, specifically containing 30–40% of the linear α-glucan amylose. Beyond starch, *Chlorella* species have a rigid cell wall, composed primarily of polysaccharides containing galactose, uronic acids, and rhamnose (Ferreira et al. 2020). These features make *C. vulgaris* a promising substrate for microbial fermentation, particularly by lactic acid bacteria (LAB), which require fermentable sugars as primary carbon sources (Abedi and Hashemi 2020; Al-Hammadi and Güngörmüşler 2024).

In fact, algal fermentation is increasingly recognised as a strategy that could enhance the nutritional and functional properties of algae, while improving their digestibility, bioaccessibility and sensory properties (Pérez-Alva et al. 2022). Particularly, lactic acid fermentation has been widely applied in food systems for thousands of years due to its capacity to improve safety, stability, and organoleptic characteristics while generating or releasing metabolites such as bacteriocins, peptides, and physically entrapped substances (Aguirre-Garcia et al. 2024). Therefore, the physicochemical transformations that occur during fermentation may enhance the health-promoting potential of microalgal biomass, thereby positioning it for use in functional foods and nutraceuticals (Garofalo et al. 2022).

However, the recalcitrant nature of the *C. vulgaris* cell wall, consisting of resistant polysaccharides and glycoproteins, poses a barrier that limits the accessibility of intracellular nutrients, thereby hindering fermentation efficiency (Spain and Funk 2022). As a result, effective pretreatments that disrupt the cell wall and release fermentable sugars are a critical step in the downstream process before further valorisation of these substrates. Towards this, various studies have focused on different pretreatments, such as microwaves (Lee et al. 2013), bead milling (Postma et al. 2017), steam explosion (Lorente et al. 2015), ultrasound-assisted hydrolysis, acid or alkali treatments, and enzymatic hydrolysis, which have been suggested to help break down its robust cell walls and facilitate the release of intracellular compounds. Nevertheless, recent reviews have highlighted the limitations of mechanical, thermal, and chemical cell disruption approaches, as economic inefficiency, sustainability concerns, and environmental impact often constrain them (Alhattab et al. 2019; Md Nadzir et al. 2023). As a result, biological approaches are increasingly favoured for their eco-friendly and cost-effective nature.

Despite growing interest in microalgal fermentation, few studies have systematically compared the effectiveness of various pretreatment techniques in enhancing fermentable sugar release from *C. vulgaris*, particularly in the context of LAB fermentation. In this study, we evaluated three pretreatment strategies: ultrasound-assisted hydrolysis and autoclave with or without acid, and enzymatic hydrolysis as strategies to enhance carbon availability from *C. vulgaris* for lactic acid fermentation. Among the various approaches, enzymatic hydrolysis yielded the best results and was performed using different setups, including Viscozyme, Alcalase, and a combination of both enzymes. Viscozyme was selected as a multi-enzyme carbohydrase complex due to its broad activity toward heterogeneous polysaccharides constituting the *C. vulgaris* cell wall, which are not efficiently degraded by single-specific carbohydrases, thereby enhancing fermentable sugar release. Alcalase was chosen for its broad proteolytic specificity and high efficiency in hydrolysing structural proteins and glycoproteins, increasing the availability of soluble peptides and amino acids. The combined application was therefore considered most competent for targeting both carbohydrate and protein fractions of the complex cell wall matrix, providing a broader substrate pool to support microbial metabolism during fermentation. We then further assessed the fermentation performance using two LAB strains, *Lactiplantibacillus plantarum* DSM 24624 and *Levilactobacillus brevis* DSM 20556. *L. plantarum* was chosen due to its broad metabolic capacity and strong adaptability to different process conditions, enabling it to efficiently and rapidly acidify the medium and produce lactic acid while *L. brevis* was selected for its heterofermentative nature, which allows it to produce diverse metabolites. Moreover, key fermentation parameters, including microbial growth, pH dynamics, and organic acid production, as well as functional properties such as antioxidant and antimicrobial activities, were monitored. Overall, this study provides a targeted evaluation of pretreatment strategies, followed by fermentation of enzyme-treated biomass variants, offering new insights into optimising microalgal substrates for functional food applications.

## Materials and methods

### Chemicals and algal biomass

The spray-dried green algae biomass powder, *C. vulgaris*, was obtained by Phytobloom (Necton S.A., Portugal). Sulfuric acid (98%), phosphoric acid (85%), ethanol (99%) commercial enzyme complex, Viscozyme (≥ 100 FBGU/g, Novozyme, Denmark), Alcalase (2.964 U mL^− 1^, Merck, Denmark), potassium sodium tartrate (KNaC_4_H_4_O_6_.4H_2_O, ACS reagent grade, ≥ 99.0%), sodium hydroxide (NaOH, ACS reagent grade, ≥ 97.0%), 3,5-dinitrosalicylic acid, D-glucose powder, bovine serum albumin, Coomassie brilliant blue G-250, 2,2′-azino-bis- (3-ethylbenzothiazolin-6-ammonium sulfonate) (ABTS), 6-hydroxy-2,5,7,8-tetramethylchromane-2-carboxylic acid (Trolox), potassium peroxodisulfate (K_2_S_2_O_8_, ACS reagent grade, ≥ 97.0%), sodium carbonate (Na_2_CO_3_, ACS reagent grade, ≥ 97.0%), sodium chloride (NaCl, ACS reagent grade, ≥ 99.0%), gallic acid (3,4,5-trihydroxybenzoic acid, 99%,), 2,2-diphenyl-1-picrylhydrazyl hydrate free radical (DPPH•, 95%), methanol, Folin–Ciocalteu reagent, copper (II) chloride solution (CuCl_2_.2H_2_O), potassium dihydrogen phosphate (KH_2_PO_4_, ACS reagent grade, ≥ 99.0%), disodium hydrogen phosphate (Na_2_HPO_4,_ ACS reagent grade, ≥ 99.0%), neocuproine (C_14_H_12_N_2_), ammonium acetate (NH_4_CH_3_CO_2,_ ACS reagent grade, ≥ 97.0%), acetonitrile (CH₃CN), formic acid (HCOOH), ammonium sulfate ((NH_4_)_2_SO_4_, ACS reagent grade, ≥ 99.0%), and MRS broth medium (De Man, Rogosa, and Sharpe) were purchased from Sigma-Aldrich (St. Louis, MO, USA).

### Proximate composition of *C. vulgaris* biomass

The protein content was measured using the Kjeldahl technique (AOAC 960.52). Moisture and ash levels were quantified through gravimetric analysis, following AOAC methods 925.10 and 900.02, respectively. Lipid content was evaluated by Soxhlet extraction with n-hexane, according to procedures previously established by our research team (Syrpas et al. 2018).

### Pretreatment of *C. vulgaris*

Pretreatment methods were assessed using a 10% (w/v) spray-dried algal biomass solution. Specifically, 7.0 ± 0.01 g of biomass was combined with 70 mL of distilled water (control) or with the specified concentrations of sulfuric acid or enzymatic solutions. All experiments were performed in triplicate.

### Ultrasound-assisted hydrolysis

Ultrasound-assisted hydrolysis was conducted using a Hielscher Ultrasonic Processor UP200Ht (Hielscher Ultrasonics, Teltow, Germany), as described previously with slight modifications (Syrpas et al. 2020). Specifically, the sonotrode operated at 90% amplitude (200 W, 26 kHz) and alternated between 10-second on and 10-second off cycles for a total duration of 30 min. Pretreatment was performed with sulfuric acid solutions at 1%, 2%, and 4% (v/v) or with distilled water as a control.

### Autoclave pretreatment

For the autoclave pretreatment, *C. vulgaris* powder was mixed with sulfuric acid at 1, 2, and 4% (v/v) or with distilled water as a control. The mixture was autoclaved at 121 °C and 1.1 atm for 30 min, then cooled to room temperature.

### Enzymatic pretreatment

Enzymatic pretreatment was carried out following conditions previously described in the literature (Mahdy et al. 2014). Specifically, samples were treated with either Viscozyme (0.3 mL g DW^− 1^ for 3 h), Alcalase (0.2 mL g DW^− 1^ for 2 h), or a combination of both (5 h total). The pH was adjusted to 5.5 for Viscozyme and 8.0 for Alcalase. Prior scouting runs at the same biomass load and temperature showed stable pH over the tested period; thus, no active pH readjustment was performed during these hydrolyses, and pH was verified at the start and end of hydrolysis (0 h and 5 h) for each replicate. After enzyme addition, samples were incubated at 50 °C in a Biosan Orbital Shaker Incubator (Latvia). Enzymes were then inactivated by heating at 75 °C for 15 min, and the samples were stored at 4 °C for further analysis.

### Preparation of bacterial inoculum

*L. plantarum* DSM 24624 and *L. brevis* DSM 20556 were obtained from Biometrija (Kaunas, Lithuania). LAB were maintained on De Man–Rogosa–Sharpe (MRS) agar slants (REF 4017282, Liofilchem, Italy), at + 4 °C in a Liebherr ProfiLine refrigerator. Inoculum preparation followed the method described previously (Aboobacker et al. 2025). Briefly, a loopful of culture was transferred from the slant to 10 mL of MRS broth (Liofilchem, Italy) and incubated at 37 °C for 16–18 h. The MRS medium was prepared by dissolving 14.04 g in 200 mL of distilled water, followed by autoclaving at 121 °C for 15 min. The resulting overnight cultures were diluted 1:100 in sterile saline to prepare working suspensions of LAB before inoculation.

### Fermentation of *C. vulgaris* with lactic acid bacteria

Prior to fermentation, 7 g of *C. vulgaris biomass* samples were pretreated with the enzymatic treatments as indicated above. The final volume of the corresponding solutions was 70 mL. Following this step, samples were inoculated with working suspensions of *L. plantarum* and *L. brevis* to achieve an initial LAB load of approximately 6 log CFU mL^− 1^, under sterile conditions. Inoculated samples were then immediately placed in a Thermo Scientific Oxoid AnaeroJar (Thermo Fisher Scientific Inc., Waltham, MA, USA) anaerostats to maintain anaerobic conditions using Oxoid AnaeroGen Compact Sachets, and incubated at 37 °C. After cell counting and pH measurements, the collected samples were centrifuged (Consul 22R, Ortoalresa—Alvarez Redondo S.A., Madrid, Spain) at 6000 rpm (4515 x g ) for 10 min, then filtered through Whatman filter paper. Samples were stored at − 18 °C in the freezer until subsequent analysis.

### Microbial growth and pH analysis

Viable cell counts in the *C. vulgaris* fermentates were determined as described previously in the literature (Dave et al. 2025). At designated time points, samples were serially diluted (1:10) in 0.9% (w/v) saline and homogenised. Dilutions were plated on MRS agar (Scharlau Chemie, Spain) and incubated at 37 °C for 36–48 h. Results were expressed as log CFU mL^− 1^.The pH of the fermented samples was monitored using a calibrated 3200P pH meter (Agilent Technologies Inc., Santa Clara, CA, USA). Measurements were taken immediately after aseptic sampling at each time point to monitor acidification during fermentation.

### Determination of reducing sugar content

The concentration of reducing sugars released during pretreatment was determined using a modified 3,5-dinitrosalicylic acid (DNS) assay, based on the method of Miller (Miller 1959) with modifications (Teixeira et al. 2012). Briefly, 1 mL of the sample was mixed with 1 mL of DNS reagent, prepared by dissolving 1 g of DNS and 30 g of sodium potassium tartrate in 100 mL of 0.4 M NaOH. Phenol was omitted from the reagent, as it can unnecessarily intensify colour. The mixture was boiled in a water bath for 5 min, then cooled to room temperature. The final volume was adjusted to 8 mL with distilled water, and absorbance was measured at 540 nm using a GENESYS 50 UV–Vis spectrophotometer (Thermo Fisher Scientific, Waltham, United States). Reducing sugar concentrations were calculated using a glucose standard curve ranging from 0.2 to 1.0 mg mL^− 1^.

### Determination of protein content

Protein content in the treated samples was determined using a modified Bradford assay (Bradford 1976). In brief, 0.1 mL of the sample was mixed with 3 mL of filtered Bradford reagent, prepared by dissolving 100 mg of Coomassie Brilliant Blue G-250 in 50 mL of 95% ethanol, followed by the addition of 100 mL of 85% (w/v) phosphoric acid, and dilution to 1 L with distilled water. After vortexing, the samples were incubated at room temperature for 10 min, then centrifuged. Absorbance of optically clear supernatants was then measured at 595 nm using a GENESYS 50 UV–Vis spectrophotometer (Thermo Fisher Scientific, Waltham, United States).

### Determination of organic acids

Organic acids were analysed in a Shimadzu LC-30AD Nexera X2 HPLC system (Shimadzu Corporation, Kyoto, Japan). Chromatographic resolution was achieved on a Rezex ROA-organic acid H+ (8%) column (300 × 7.8 mm, Torrance, CA, USA), maintained at 60 °C, using 0.005 M sulfuric acid as the mobile phase under isocratic conditions, at a flow rate of 0.5 mL min^− 1^. Qualitative determination was performed using reference materials, whereas quantitative determination was performed using external calibration curves. The final results were expressed as mg mL^− 1^ using dose-response curves for lactic acid (0.1-5 mg mL^− 1^, y = 63740x − 6516.5, R² = 0.9979, LOD: 0.026 g L^− 1^; LOQ: 0.078 g L^− 1^), acetic acid (0.1 -5 mg mL^− 1,^ y = 54906x − 748.81, R² = 0.9999, LOD: 0.030 g L^− 1^; LOQ: 0.091 g L^− 1^).

### Total phenolic content (TPC) by Folin-Ciocalteu’s assay

TPC of *C. vulgaris* supernatants was determined using the method of Singleton (Singleton et al. 1999) with modifications reported (Nagybákay et al. 2023). For each analysis, 150 µL of the fermented extract or distilled water (blank) was mixed with 750 µL of Folin–Ciocalteu reagent (diluted 1:9, v/v). After 3 min of reaction, 600 µL of a sodium carbonate solution (75 g L^− 1^) was added, and the mixture was vortexed for 15 s. The samples were then incubated in the dark for 2 h. Absorbance was measured at 760 nm. TPC was expressed as milligrams of gallic acid equivalents (mg GAE g DW⁻¹), based on a standard curve prepared with gallic acid (0–80 µg mL^− 1^).

### Cupric reducing antioxidant capacity assay

CUPRAC measurements were performed according to the protocol of Apak (Apak et al. 2007). Briefly, 0.4 mL of Cu(II), neocuproine, and NH_4_Ac buffer solutions were added to a test tube. Then, 0.4 mL of extract (or standard) was added to the initial mixture. The tubes were stoppered, and after 30 min, the absorbance was recorded at 450 nm. The CUPRAC antioxidant capacity was expressed as Trolox equivalents (mg Trolox g DW⁻¹), employing a dose-response curve for Trolox (25–150 µmol L^− 1^).

### Determination of the ABTS^·+^ scavenging assay

The ABTS assay was carried out according to the method described in the literature (Re et al. 1999). Firstly, phosphate-buffered saline (PBS) solution (75 mmol L^− 1^; pH 7.4) was prepared by dissolving 8.18 g of NaCl, 0.27 g of KH_2_PO_4_, 1.42 g of Na_2_HPO_4_, and 0.15 g of KCl in 1 L of ultra-pure water. The ABTS^·+^ solution was prepared by mixing 50 mL of ABTS (2 mmol L^− 1^ PBS) with 200 µL of K_2_S_2_O8 (70 mmol L^− 1^) and allowing the mixture to stand in the dark at room temperature for 15–16 h prior to use. The working solution was prepared by diluting the ABTS^·+^ solution with PBS to achieve an absorbance of 0.700 ± 0.010 at 734 nm. To a 1500 µL working ABTS^·+^ solution, 25 µL of fermented extract or blank (methanol) was added. The mixtures were kept in the dark for 2 h, and the absorbance was measured at 734 nm. The final results were expressed as Trolox equivalents (TEAC mg Trolox g DW⁻¹), calculated employing dose-response curves for Trolox (0–1500 µmol L^−¹^ methanol).

### Determination of the DPPH scavenging assay

The DPPH radical scavenging assay was performed as previously described (Brand-Williams et al. 1995). To 500 µL of fermented extract or methanol (blank), 1000 µL of a ~ 89.7 µmol L^− 1^ (absorbance adjusted to 0.800 ± 0.010 AU at 517 nm) DPPH· methanolic solution was added and vortexed for 15 s, then kept for 2 h in the dark, and the absorbance of the optically clear supernatant was measured at 517 nm. Antioxidant activity was expressed as Trolox equivalents (TEAC mg Trolox g DW⁻¹), calculated using dose-response curves for Trolox (0–50 µmol L^− 1^ methanol).

### Determination of antimicrobial activity

The antibacterial activity of the samples was evaluated using the agar well diffusion method against reference strains: *Escherichia coli* ATCC 8739, *Micrococcus luteus* ATCC 9341, *Staphylococcus aureus* ATCC 25923, *Bacillus subtilis* ATCC 6633, and *Pseudomonas aeruginosa*. To prepare the bacterial inoculum, each strain was cultured on Plate Count Agar slants at 37 °C for 18–20 h. A loopful of the culture was transferred into sterile saline and adjusted to a 0.5 McFarland standard, corresponding to approximately 10⁸ CFU mL^− 1^. Then, 20 mL of this suspension was mixed with 200 mL of molten Plate Count Agar, poured into Petri dishes, and allowed to solidify. Wells (9 mm diameter) were created using sterile pipette tips, and 25 µL of either unfermented control or 24-hour fermented samples was added to each well. Plates were incubated at 37 °C for 24 h, after which the diameters of inhibition zones were measured in millimetres using an electronic digital calliper. Supernatants were not neutralised or treated with catalase or proteases; hence, the results represent screening-level inhibition under native matrix conditions.

### Statistical analysis

Mean values and standard deviations were calculated using Microsoft Excel. All experiments were performed using biological triplicates of clarified supernatants, which were centrifuged, filtered, and freeze-dried prior to analysis (*n* = 3). Each replicate corresponds to an independent experimental sample. One-way ANOVA followed by Tukey’s post hoc test was conducted to compare means showing significant variation (*p* < 0.05), using GraphPad Prism software version 10.5 for Windows.

## Results and discussion

### Compositional analysis of *C. vulgaris*

In the first part of this study, the proximate composition of *C. vulgaris* biomass was analysed, with the results presented in Table 1. As shown, protein (~ 46%) and carbohydrates (~ 40%) were the primary components of the studied biomass. In comparison, lipid (~ 3%) and ash (~ 6%) contents were lower (Table 1). These findings are consistent with a recent study, which reported ash, lipid, protein, and carbohydrate contents of approximately 9%, 0.5%, 40%, and 43%, respectively (Damayanti et al. 2025). Furthermore, previous reviews reported that the total protein content in mature *C. vulgaris* ranges from 42% to 58% of the biomass dry weight (Safi et al. 2014; Ijaola et al. 2024). Although carbohydrate levels are typically lower, *Chlorella* is known to accumulate high starch contents, particularly under nitrogen-limiting conditions, reaching a total carbohydrate content of up to 55% of its dry weight (Brányiková et al. 2011). The observed lipid content in this study is lower than most reported values in the literature, ranging from 5% to 58%, with higher content typically obtained under mixotrophic cultivation (Wang et al. 2024). Nevertheless, the biochemical and nutritional profile of *C. vulgaris* is highly dependent on cultivation conditions and the strain used (Hiltunen et al. 2026).

**Table 1:** Proximate composition of *C. vulgaris*

Parameters	*C. vulgaris*
Moisture (%)	5.19 ± 0.01
Total ash (%)	5.78 ± 0.02
Crude protein (%)	46.34 ± 0.14
Crude lipid (%)	3.19 ± 0.03
Carbohydrate (%)	39.50 ± 0.14

Results represent means ± SD (*n* = 3)

### Evaluation of various pretreatment strategies

**Fig. 1:**
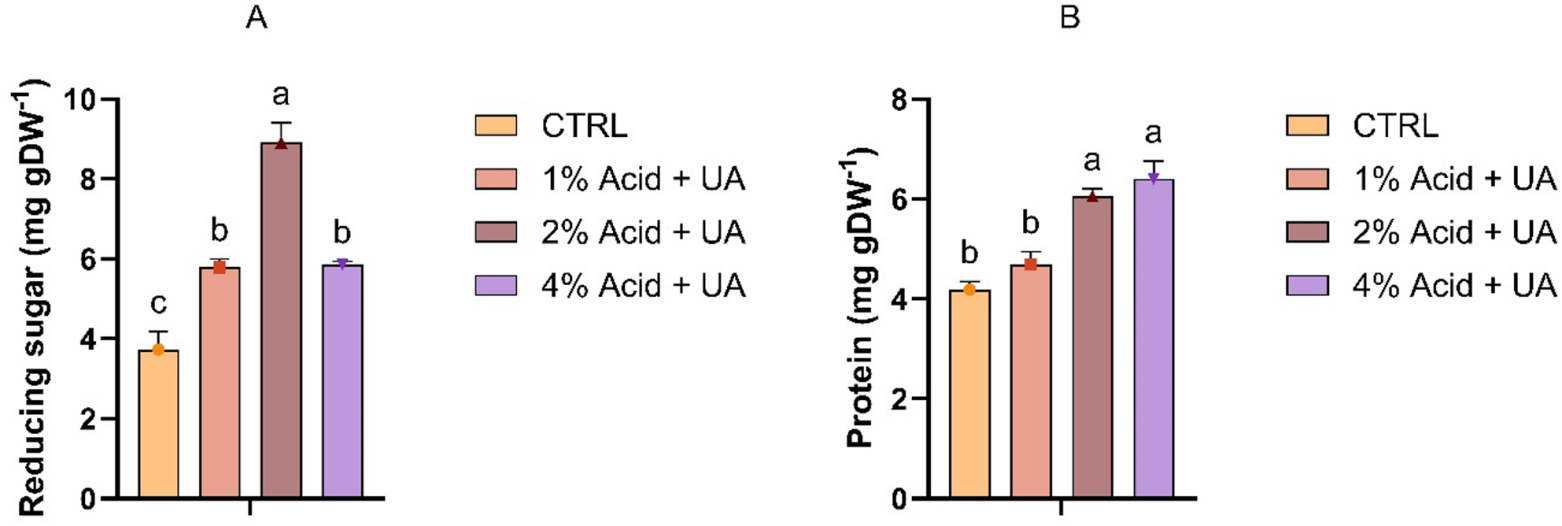
Reducing sugar (**A**) and protein content (**B**) after ultrasound treatments (UA) with various concentrations of sulfuric acid. Values are presented as means ± SD. Different letters indicate statistically significant differences (*p* < 0.05) (*n* = 3)

The first method used to disrupt the cell wall of *C. vulgaris* was ultrasound-assisted hydrolysis, performed in distilled water or sulfuric acid solutions at varying concentrations (Fig. 1). Ultrasound-assisted extraction is a green technology for recovering bioactive compounds from algae, with reduced solvent and energy input while preserving molecular integrity. Ultrasound induces cavitation in liquid media, and the collapse of these microbubbles generates localised mechanical forces that disrupt the cell walls of microalgae, facilitating the release of intracellular compounds (Lee et al. 2017). As a technique, it has been widely applied to extract polysaccharides, pigments, and phenolics from various algal species (Carreira-Casais et al. 2021). The highest release of reducing sugars (~ 9 mg g DW^− 1^) was achieved with 2% sulfuric acid (Fig. 1A), while the maximum protein content was observed with 4% acid. However, no statistically significant difference was observed when compared with 2% acid (Fig. 1B). As shown, even at the highest yield achieved with this method, the carbohydrate conversion remained relatively low at 2.2%. This result closely agrees with a previous study utilising ultrasound-assisted acid hydrolysis for *Chlorella*, which reported a carbohydrate conversion efficiency of 3.3%. The authors concluded that the application of ultrasonic treatments as the only method for releasing and depolymerising carbohydrates is insufficient (Nasirpour et al. 2023). Although ultrasound treatments are a useful pretreatment method in other applications, they may not achieve sufficient conversion of complex carbohydrates into simple sugars, which are essential for subsequent fermentation.

**Fig. 2:**
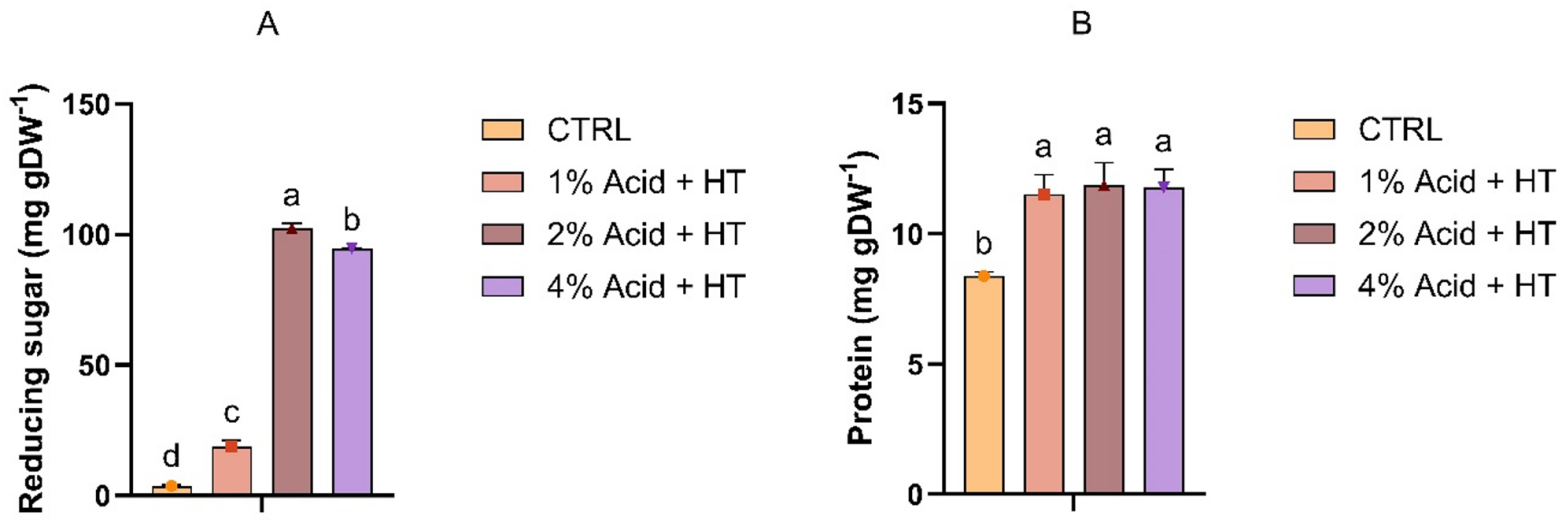
Reducing sugar (**A**) and protein concentration (**B**) after hydrothermal treatment (HT). Bars represent means ± SD. Different letters indicate statistically significant differences (*p* < 0.05) (*n* = 3)

In the following step, hydrothermal hydrolysis was conducted at 121 °C for 30 min using sulfuric acid concentrations of 1%, 2%, and 4% (v/v), with distilled water serving as the control. The results, summarised in Fig. 2, indicate that hydrolysis with 2% sulfuric acid yielded the highest amount of reducing sugars, reaching 102.4 mg g DW^− 1^ with a conversion efficiency of 25.9% (Fig. 2A). On the other hand, the lowest yield was observed for hydrothermal treatment with water as solvent, reaching a conversion efficiency of only 0.9% (3.7 mg g DW^− 1^). A similar observation was reported in a study on *Chlorella*, where the conversion efficiencies at 30 and 120 min of heating in the hydrothermal method were similarly low, at 0.5% and 1%, respectively (Nasirpour et al. 2023). Protein recovery was significantly higher in samples treated with 2% and 4% acid than in those treated with lower concentrations. However, the difference between the two highest concentrations was not statistically significant (Fig. 2B). The lack of significant improvement in protein recovery at higher acid concentrations may be due to structural changes in the proteins, i.e., the release of short peptides, induced by stronger acid hydrolysis (Dent et al. 2024).

Regardless of the technique used, the addition of sulfuric acid resulted in a substantially higher release of reducing sugars and proteins (Figs. 1 and 2). Dilute sulfuric acid is commonly employed for lignocellulosic biomass pretreatment due to its rapid reaction kinetics and enhanced cellulose hydrolysis efficiency (Esteghlalian et al. 1997; Sun and Cheng 2002). In practically all cases, a 2% sulfuric acid addition resulted in the highest recoveries. This observation aligns with a report by de Carvalho Silvello et al. (2023), who investigated the effect of 1%, 2.5%, and 4% sulfuric acid concentrations on the release of sugars from biomass. The authors indicated that 2.5% sulfuric acid yielded the highest sugar release, while increasing the concentration to 4% did not result in a significant improvement (de Carvalho Silvello et al. 2023). Moreover, in another study, the effects of 2% and 5.36% sulfuric acid at 120 °C for 30 min on sugar release were compared, with the authors reporting that the highest yield (247 mg g DW^-1^) was achieved with the 2% acid concentration (Abou El-Souod et al. 2021). Nevertheless, it is worth noting that a previous study comparing five acids for *C. vulgaris* hydrolysis found that while hydrochloric, sulfuric, and nitric acids were more effective than peracetic and phosphoric acids, hydrochloric acid yielded the best overall results (Park et al. 2016). High recoveries (> 80%) have also been reported by other studies utilising hydrochloric acid for the pretreatment of *Chlorella* biomass towards bioethanol production (Kim et al. 2011; Zhou et al. 2011). Nonetheless, during acid hydrolysis, especially at elevated temperatures, carbohydrate degradation often leads to the formation of furanic compounds, such as hydroxymethylfurfural and furfural, which are known to inhibit microbial activity and reduce fermentation efficiency (Labbafi et al. 2025).

**Fig. 3:**
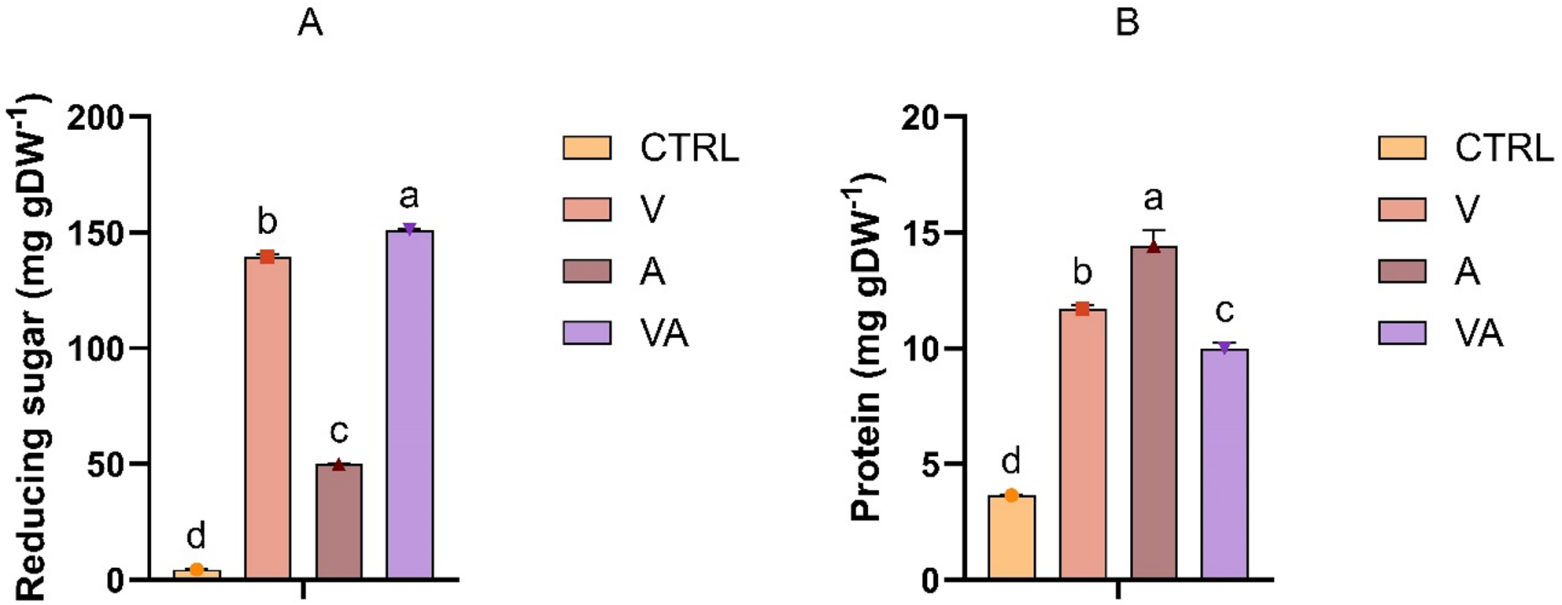
Reducing sugar (**A**) and protein concentration (**B**) after enzymatic hydrolysis (V: Viscozyme treated; A: Alcalase; VA: Viscozyme-Alcalase). Bars represent means ± SD. Different letters indicate statistically significant differences (*p* < 0.05) (*n* = 3)

Considering these factors, the next step was to apply three enzymatic treatments to disrupt the cell wall matrix and enhance the release of intracellular compounds. Enzymatic hydrolysis has been proposed as an effective method to disrupt the cell wall of *C. vulgaris*, thereby facilitating the release of valuable intracellular components, including proteins, carbohydrates, and bioactive compounds (Rahman et al. 2022). Towards this, Viscozyme, a multi-enzyme complex with carbohydrase activity, was used to break down polysaccharides, while Alcalase, a protease, targeted protein structures. Additionally, a combined treatment using both enzymes was performed to maximise cell wall degradation and improve nutrient availability for downstream processes. As shown in Fig. 3A, enzymatic hydrolysis, particularly with Viscozyme alone and in combination, significantly increased the release of reducing sugars, yielding 139.61 and 151.18 mg g DW^− 1^, with the conversion efficiency of 35.3% and 38.3%, respectively, compared to 4.34 mg g DW^− 1^, where the efficiency reached only 1.1%, in the control samples. These findings are in close agreement with a previous report, which reported that the highest soluble carbohydrate hydrolysis (160 mg g DW^− 1^) was obtained with the carbohydrolase (V and V + A) after 2 h (Mahdy et al. 2014). On the other hand, Alcalase treatment resulted in the highest protein release, reaching 14.4 mg g DW^− 1^, corresponding to an extraction efficiency of 3.1%. Similar recovery values were reported in a previous study, where the enzymatic protein extraction process yielded an average efficiency of 3.80% (Yaghoubzadeh and Safari 2025).

Overall, among the various tested techniques, ultrasound resulted in the lowest recovery of fermentable sugars and protein, followed by the hydrothermal treatments. The low efficiency of ultrasound has also been reported in previous studies. For instance, de Carvalho et al. evaluated alkaline, acid, ultrasonic, and supercritical treatments and found that ultrasound in water (10 min, 200 W, 20 kHz) yielded less than 1 g L^−1^of glucose, one of the lowest values among the tested methods (de Carvalho Silvello et al. 2023). Similarly, Paterson et al. applied ultrasound after freeze–thaw cycles, but microscopy revealed insufficient cell wall disruption, highlighting the need for more effective strategies (Paterson et al. 2024). Hydrothermal treatment also did not result in satisfactory results in this study. However, it should be noted that a previous report indicated that although the hydrothermal or ultrasonic treatment alone may be insufficient, their combination results in substantially higher recoveries of fermentable sugars (Nasirpour et al. 2023). The effectiveness of combinatorial approaches has also been reported in other studies, where alkali and enzyme hydrolysis yielded the highest reducing sugar yield (Effiom et al. 2023).

In this study, enzymatic treatments resulted in substantially higher recoveries of fermentable sugars, especially as compared to the other two methods. The efficiency of cellulases and hydrolases in converting the complex carbohydrates of *Chlorella* into fermentable sugars has been demonstrated for biohydrogen (Liu et al. 2021; Sriyod et al. 2021) and bioethanol (Kim et al. 2014). In fact, a recent review concluded that compared to other pretreatment methods, enzymatic hydrolysis offers advantages for bioethanol production due to its low cost, minimal environmental impact, and higher ethanol yields, as it also operates under mild conditions, requiring less energy and no harsh chemicals, thereby avoiding the formation of fermentation inhibitors (Kusmiyati et al. 2023). Besides bioenergy production, enzymatic hydrolysis has also been used to enhance the functional properties of *Chlorella* hydrolysates (Yin et al. 2010; Gharehbeglou et al. 2024). An additional benefit of this approach is the absence of fermentation inhibitors. Considering all these factors, enzyme-assisted hydrolysis was selected as the optimal strategy prior to LAB fermentation, as discussed in the following sections.

### Fermentation of *C. vulgari*s by lactic acid bacteria

#### Microbial growth and acidification during LAB fermentation

**Fig. 4:**
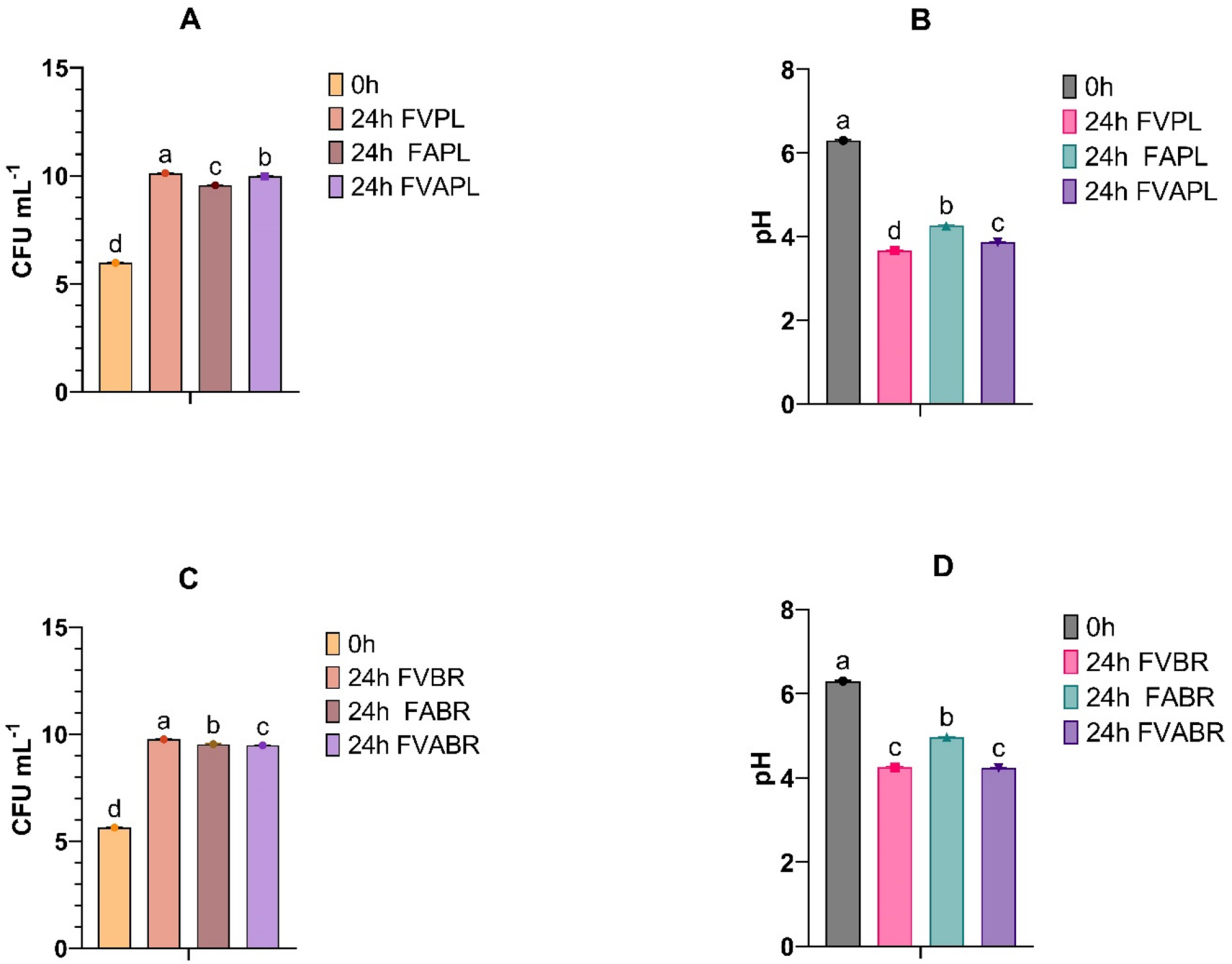
Microbial growth of *L. plantarum* (**A**) and *L. brevis* (**B**) and pH of *L. plantarum* (**C**) and *L. brevis* (**D**) during 24-hour fermentation. (FVPL, FVBR: Viscozyme treated and fermented with *L. plantarum* and *L. brevis*, respectively; FAPL, FABR: Alcalase treated and fermented with *L. plantarum* and *L. brevis*, respectively; FVAPL, FVABR: Combined enzyme treated and fermented with *L. plantarum* and *L. brevis*, respectively). Bars represent means ± SD. Different letters indicate statistically significant differences (*p* < 0.05) (*n* = 3)

In this part of the study, *C. vulgaris* biomass underwent enzymatic hydrolysis prior to fermentation by LAB. To evaluate the effectiveness of the fermentation process and the capacity of pretreated biomass to support microbial activity, the growth of inoculated LAB strains was monitored and expressed as log CFU mL⁻¹. Concurrently, pH was monitored to assess acidification of the fermentation medium. The results of these analyses are summarised in Fig. 4. Before inoculation, the cell densities of *L. plantarum* and *L. brevis* were standardised to approximately 6 log CFU mL⁻¹ to ensure consistent initial microbial loads across all fermentation trials. As depicted in Fig. 4, both strains exhibited substantial growth after 24 h of fermentation. In samples fermented with *L. plantarum*, the highest growth of 10.12 log CFU mL⁻¹, corresponding to a Δ log CFU mL⁻¹ of 4.1, was observed for the sample treated with Viscozyme (FVPL, Fig. 4A). The other two treatments, FAPL and FVAPL, also showed a significant increase in cell density with Δ log CFU mL⁻¹ of 3.6 and 4.0, respectively, thereby indicating robust proliferation under the given conditions (Fig. 4A).

Similar trends were observed in samples inoculated with *L. brevis*, with all treatments supporting effective growth. The Viscozyme-treated samples (FVBR) reached the highest cell density of 9.76 log CFU mL⁻¹, with Δ log CFU mL⁻¹ of 4.1 (Fig. 4C). The other treatments also showed strong LAB proliferation, with increases of ~ 3.9 log units.

In addition to microbial growth, acidification of the medium was used as an indicator of fermentation efficiency. As illustrated in Fig. 4B and D, both LAB strains demonstrated significant acidification capacity within 24 h of fermentation. The lowest pH values were recorded in the Viscozyme-treated samples, reaching 3.6 for *L. plantarum* (Fig. 4B) and 4.2 for *L. brevis* (Fig. 4D). In contrast, the lowest acidification for both strains was observed in the Alcalase-treated samples.

These findings align with previous studies reporting the beneficial effects of *Chlorella* species on the viability and growth of probiotic strains, such as *Lactobacillus* and *Bifidobacterium.* In most cases, *Chlorella* biomass was incorporated into fermented semi-solid dairy matrices, such as cheese and yoghurt (Kovaleski and Ventura 2025), or into freeze-dried probiotic formulations (Meireles Mafaldo et al. 2022; Fortuin et al. 2026). For instance, Ścieszka and Klewicka demonstrated that supplementing MRS medium with varying concentrations of *C. vulgaris* biomass accelerated the growth of four *L. brevis* strains, shortening their logarithmic growth phase (Ścieszka and Klewicka 2020). Moreover, another study reported that *C. vulgaris* increased the survival of Lactobacillus spp. in bile salts. They noted that the protective effect of algae at low pH is strain-dependent, concluding that the biomass of this microalga could be used in fermented dietary products (Sylwia and Elżbieta 2020).

However, only a limited number of studies have explored the use of *C. vulgaris* biomass as a sole carbon source for LAB fermentation. In a recent study, Niocolloti et al. investigated the fermentation of four *C. vulgaris* biomasses with varying carbohydrate content using 5 LAB strains, including *L. plantarum* 4932. Their results showed that all biomasses fermented with *L. plantarum* reached pH values below 4.5 after 72 h of fermentation, although microbial growth was modest, reaching a maximum of 2.1 Δ log CFU mL⁻¹ (Nicolotti et al. 2025). Moreover, another study reported co-fermentation of *Aphanizomenon flos-aquae* and *C. vulgaris* biomasses with *L. plantarum* and *S. cerevisiae*; however, the authors do not report cell growth or pH values (Tomassi et al. 2025). Taken together, the results of the present study highlight the importance of enzymatic pretreatment in enhancing the fermentability of *C. vulgaris* biomass. Compared to previous reports, the observed microbial proliferation and acidification suggest that enzymatic hydrolysis significantly improves the bioavailability of fermentable substrates, thereby supporting more efficient LAB growth and metabolic activity.

#### Analysis of sugars and organic acids

**Fig. 5:**
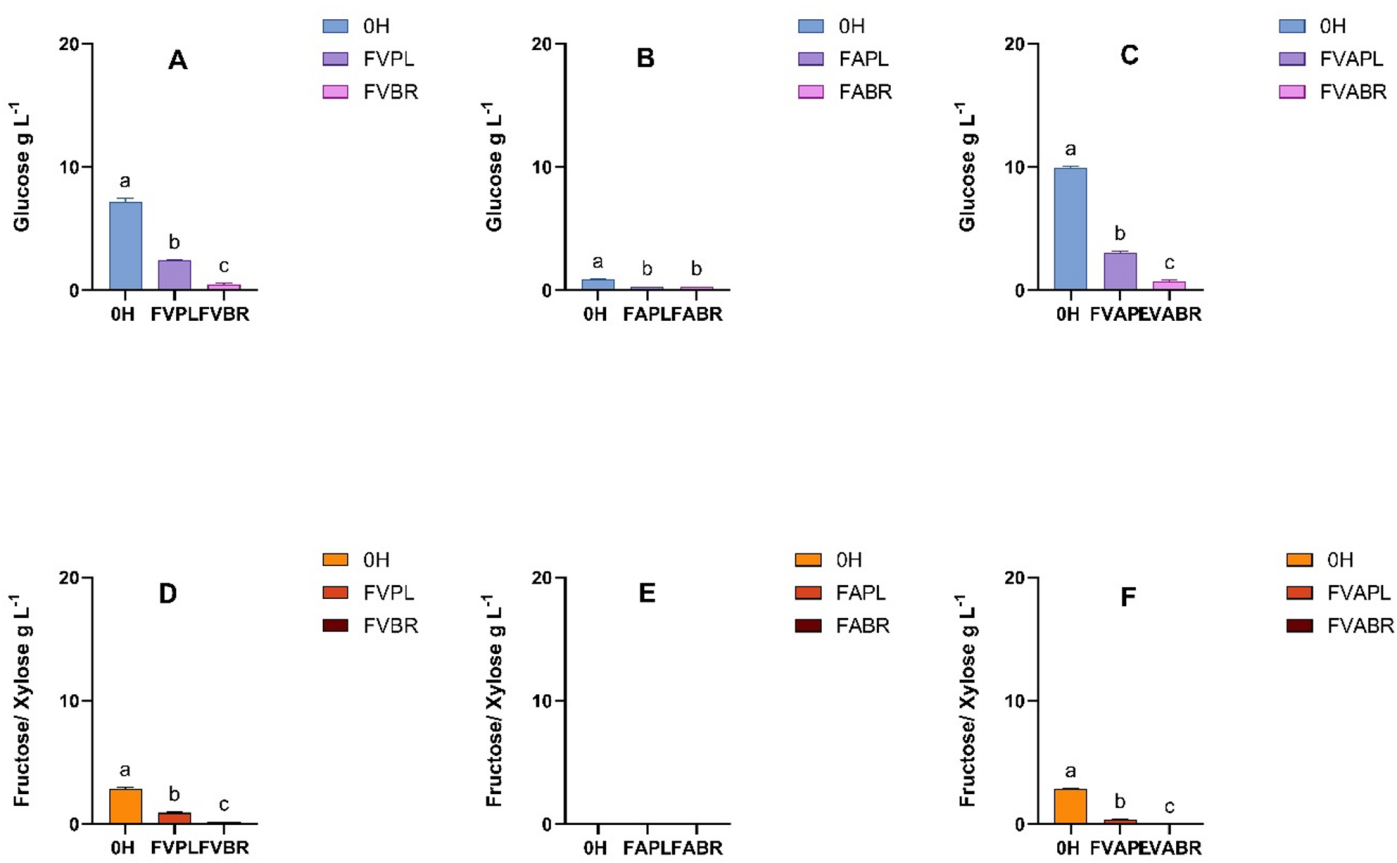
Glucose (**A**–**C**) and fructose/xylose (**D**–**F**) concentration after fermentation with *L. plantarum* (PL*) and L. brevis (BR). (0 H*: Unfermented; FVPL, FVBR: Viscozyme treated and fermented with *L. plantarum* and *L. brevis*, respectively; FAPL, FABR: Alcalase treated and fermented with *L. plantarum and L. brevis*, respectively; FVAPL, FVABR: Combined enzyme treated and fermented with *L. plantarum and L. brevis*, respectively). Bars represent means ± SD. Different letters indicate statistically significant differences (*p* < 0.05) (*n* = 3)

In parallel with the growth experiments, the reducing sugar content and organic acid production were monitored, with results summarised in Fig. 5. Generally, *L. plantarum* exhibits facultative heterofermentative metabolism, meaning its fermentation products can differ depending on the environmental conditions (Siezen and van Hylckama Vlieg 2011). Most LAB thrive in anaerobic environments, where they convert pyruvate produced via the Embden-Meyerhof-Parnas pathway into lactate. On the other hand, *L. brevis* is an obligate heterofermentative LAB, as it consistently produces a mixture of lactic acid, carbon dioxide, and ethanol or acetic acid during fermentation, regardless of culture conditions. Unlike facultative fermenting bacteria, it relies on the phosphoketolase pathway to metabolise sugars. A significant reduction in reducing sugar concentration was observed after 24 h of fermentation (Supplementary Material Fig. S1). Compared with Al calase-treated samples, sugar consumption was higher in Viscozyme-treated and combined-treatment samples. Chromatographic analysis confirmed that the hydrolysates primarily contained glucose, fructose, and xylose, along with maltose (Fig. 5 and Supplementary Material Fig. S2). Due to chromatographic limitations, fructose and xylose were not fully resolved. These findings are consistent with previous reports on the carbohydrate composition and enzymatic hydrolysis of *C. vulgaris*, which typically release glucose, fructose, xylose, and maltose (Safi et al. 2014; Mahdy et al. 2014; El-Naggar et al. 2020). Both LAB strains exhibited similar overall sugar consumption, except in Alcalase-treated samples, where *L. plantarum* consumed more sugars than *L. brevis*. Xylose utilisation was greater in *L. brevis*, aligning with its metabolic capacity to ferment pentoses via the phosphoketolase pathway (Zhang et al. 2016). This pathway also explains the small amounts of acetic acid detected in samples fermented by *L. brevis* (Supplementary Fig. S3).

**Fig. 6:**
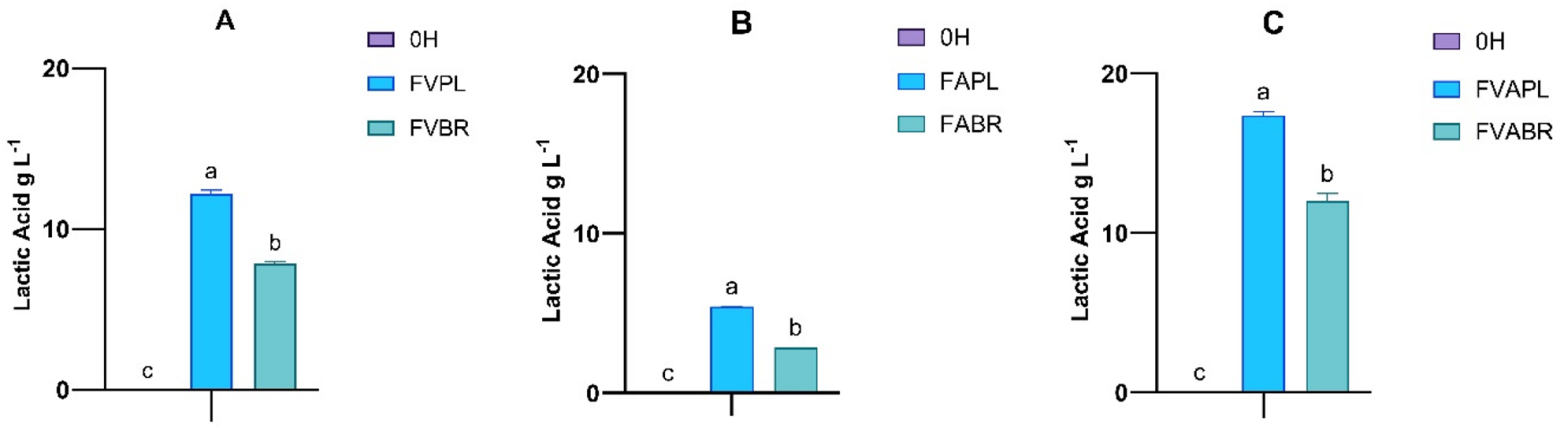
Lactic acid (**A**–**C**) concentration after fermentation with *L. plantarum (PL)* and *L. brevis (BR).* (0 H: Unfermented; FVPL, FVBR: Viscozyme treated and fermented with *L. plantarum* and *L. brevis*, respectively; FAPL, FABR: Alcalase treated and fermented with *L. plantarum and L. brevis*, respectively; FVAPL, FVABR: Combined enzyme treated and fermented with *L. plantarum* and *L. brevis*, respectively). Bars represent means ± SD. Different letters indicate statistically significant differences (*p* < 0.05) (*n* = 3)

Correspondingly, the production of organic acid is presented in Fig. 6. The sample treated with combined enzymes and fermented with *L. plantarum* exhibited the highest lactic acid production among all tested conditions, at 17.35 g L^− 1^, followed by the sample treated with Viscozyme alone, at 12.20 g L^− 1^. These findings align with the measured reducing sugars, as Viscozyme- and combined-treated samples showed higher sugar release from the matrix, confirming the increased conversion to lactic acid. In addition, a small concentration of acetic acid production was observed in the samples, including those treated with Alcalase and combined enzyme treatments, and fermented by *L. brevis*, with values of 1.03 and 1.10 g L^− 1^, respectively (Supplementary Materials Fig. S3).

The enhanced fermentation performance observed in Viscozyme and combined enzyme treatments further highlights the importance of cell wall disruption in microalgae. Several reports on enzymatic pretreatment of *Chlorella* species for biofuel and bioproduct generation have shown similar benefits, including increased sugar release and improved microbial activity (Córdova et al. 2019; Yang et al. 2023). In a previous study, the authors explored fermentation strategies for lactic acid production with immobilised *L. plantarum* 23 using *C. vulgaris* hydrolysate as feedstock. They tested various aeration conditions to determine the oxygen requirements for this strain to be homolactic (Chen et al. 2020). The study concluded that although lactic acid’s yield and productivity were not substantially different under different oxygen availability conditions, the strain was homolactic only under anaerobic conditions (Chen et al. 2020). In another study, the feasibility of *C. vulgaris* as a feedstock for lactic acid production was investigated (Tu et al. 2026). After sulfuric acid hydrolysis, fermentation of the hydrolysate with *L. casei* produced 10.7 g/L lactic acid within 24 h. Another study investigated the effect of untreated *C. vulgaris* on fermentation dynamics, i.e., acidifying activity, of different LAB strains (Ścieszka and Klewicka 2020). The tested strains produced L-lactic acid (ranging from 6.95 to 8.15 g/L). It was concluded that *C. vulgaris* helped improve the L-lactic acid production. These studies have demonstrated the feasibility of *C. vulgaris* for lactic acid production. However, in our study, enzymatic hydrolysis proved more effective at breaking down microalgal biomass into fermentable sugars, resulting in a significantly higher lactic acid production yield. A comprehensive summary of the fermentation performance, including sugar concentration, lactic acid production, productivity, Δ log growth, and final pH, is presented in Table 2.

**Table 2:** Summary of fermentation outcomes

Time	Samples	Sugars (g/L)	Productivity (g/L·h)	Lactic acid (g/L)	Final pH	Δlog CFU
0 h	V	14.25 ± 0.08	0.00 ± 0.00	0.00 ± 0.00	4.94 ± 0.02	-
	A	5.29 ± 0.01	0.00 ± 0.00	0.00 ± 0.00	5.23 ± 0.01	-
	VA	15.40 ± 0.06	0.00 ± 0.00	0.00 ± 0.00	5.16 ± 0.01	-
24 h	FVPL	3.17 ± 0.03	0.51 ± 0.01	12.2 ± 0.20	3.67 ± 0.01	4.15 ± 0.02
	FAPL	1.24 ± 0.01	0.23 ± 0.01	5.41 ± 0.02	4.26 ± 0.02	3.59 ± 0.02
	FVAPL	3.72 ± 0.25	0.72 ± 0.01	17.35 ± 0.24	3.86 ± 0.01	4.01 ± 0.02
	FVBR	2.38 ± 0.02	0.33 ± 0.01	7.83 ± 0.11	4.25 ± 0.02	4.12 ± 0.01
	FABR	2.0 ± 0.06	0.12 ± 0.01	2.83 ± 0.01	4.97 ± 0.01	3.89 ± 0.03
	FVABR	3.36 ± 0.12	0.5 ± 0.01	11.99 ± 0.45	4.23 ± 0.02	3.84 ± 0.01

Results represent mean ± SD (0H: Unfermented; V, A, VA: Viscozyme, Alcalase and combined treatment; FVPL, FVBR: Viscozyme treated and fermented with *L. plantarum* and *L. brevis*, respectively; FAPL, FABR: Alcalase treated and fermented with *L. plantarum* and *L. brevis*, respectively; FVAPL, FVABR: Combined enzyme treated and fermented with *L. plantarum* and *L. brevis*, respectively)

Nevertheless, the ability of *C. vulgaris* to serve as a sole carbon source for LAB fermentation, especially when pretreated enzymatically, could open new avenues for sustainable bioprocessing. This strategy could also be considered particularly relevant for developing functional foods and probiotic formulations, as highlighted by recent work on engineered *C. vulgaris* strains with enhanced carbohydrate content (Saha et al. 2024).

### Antioxidant activity of fermented *C. vulgaris*

**Fig. 7:**
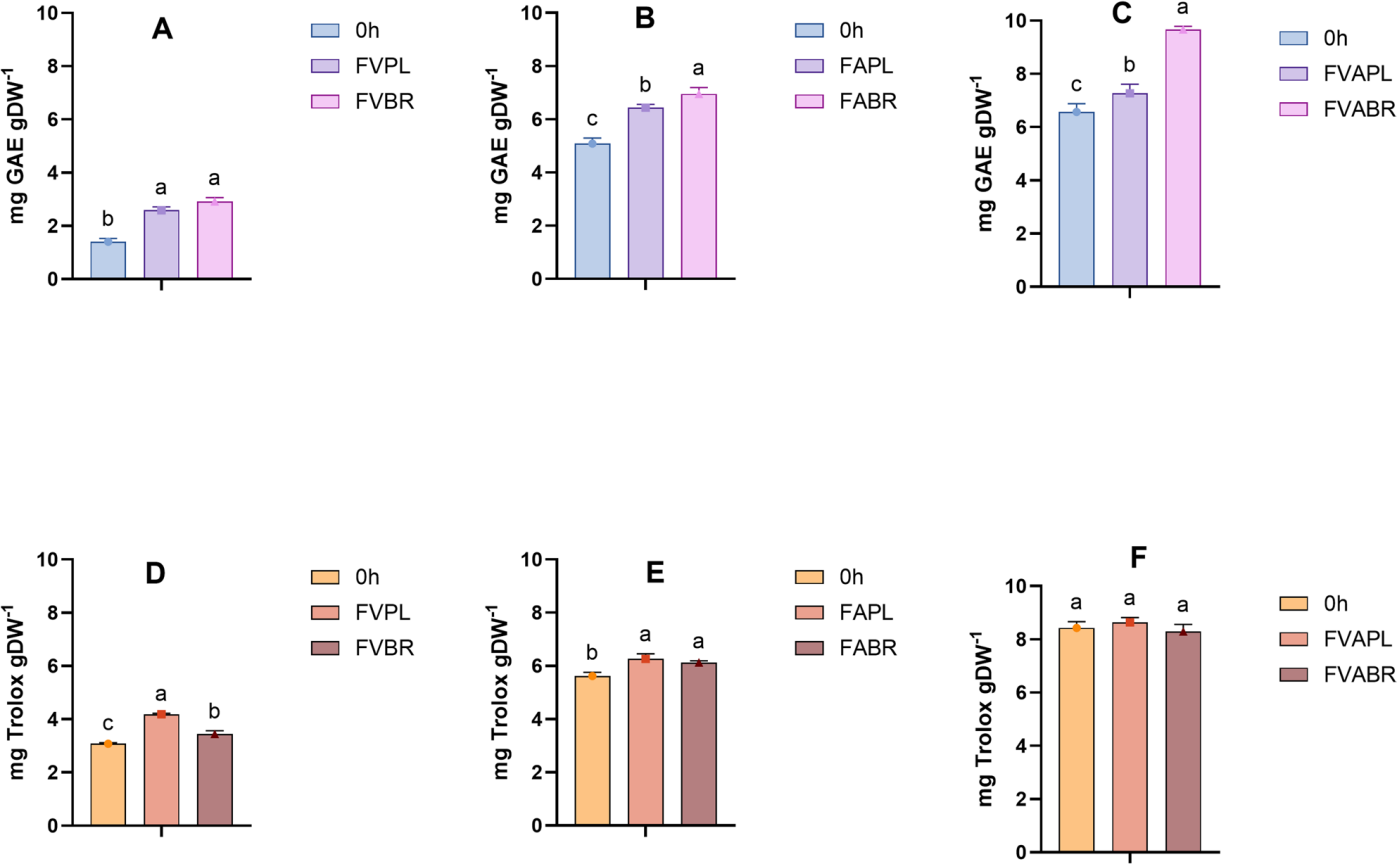
In vitro antioxidant capacity of *C. vulgaris* as evaluated by the total phenolic content (**A**–**C**), and CUPRAC (**D**–**F**) assay after fermentation with *L. plantarum* (PL) and *L. brevis (*BR). (0 H: Unfermented; FVPL, FVBR: Viscozyme treated and fermented with *L. plantarum* and *L. brevis*, respectively; FAPL, FABR: Alcalase treated and fermented with *L. plantarum* and *L. brevis*, respectively; FVAPL, FVABR: Combined enzyme treated and fermented with *L. plantarum* and *L. brevis*, respectively). Bars represent means ± SD. Different letters indicate statistically significant differences (*p* < 0.05) (*n* = 3)

The antioxidant capacity of *C. vulgaris* biomass and its extracts is primarily associated with a diverse range of bioactive compounds, including chlorophyll, carotenoids (such as lutein and β-carotene), polysaccharides, and polyphenols (Mendes et al. 2024). Although fermentation has been proposed as a strategy to enhance the antioxidant activity of microalgae, data on its impact on *C. vulgaris* and its extracts remain limited. In this study, the in vitro antioxidant capacity of fermented *C. vulgaris* was assessed using four complementary assays: CUPRAC, total phenolic content (TPC), ABTS, and DPPH radical scavenging tests (Fig. 7 and Supplementary Material Fig. S4). Enzyme-treated but non-fermented samples served as controls (0 h in all figures). Across all assays, combined enzyme treatment yielded the highest antioxidant capacity, with values of 8.64 mg Trolox g DW⁻¹ (CUPRAC), 9.67 mg GAE g DW⁻¹ in the TPC assay (Fig. 7). Similar observations with 19.46 mg Trolox g DW⁻¹ in the ABTS assay, and 1.45 mg Trolox g DW⁻¹ in the DPPH assay were recorded (Supplementary Material Fig. S4). However, most differences between fermented and enzyme-treated samples were not statistically significant.

When a single enzyme pretreatment was applied, the effects of fermentation became more evident, with fermented samples generally exhibiting higher antioxidant activity than unfermented ones, with the greatest increase in TPC from 1.40 to 2.90 mg GAE g DW⁻¹. Among other assays, the increases were 36.2%, 9%, and 38.75% for CUPRAC, ABTS, and DPPH, respectively. This observation aligns with recent findings by Tomassi et al., who reported that fermentation of *C. vulgaris* using LAB and yeast significantly improved its antioxidant profile. Specifically, TPC nearly doubled from 2.4 mg GAE g DW⁻¹ at the onset of fermentation to 4.8 mg GAE g DW⁻¹ after 24 h, with similar trends observed for DPPH and ORAC assays (Tomassi et al. 2025).

The increase in antioxidant capacity observed in this study can be attributed to several mechanisms. Enzymatic pretreatment with cellulases (e.g., Viscozyme) facilitates the release of intracellular antioxidants, including phenolics, carotenoids, chlorophylls, and peptides (Sanjeewa et al. 2023; Badrunanto et al. 2025). Moreover, although proteases can contribute to cell wall disruption, the observed increase in antioxidant capacity is attributed to the generation of bioactive peptides with antioxidant properties. These observations are further supported by previous studies on other substrates that have shown similar effects of proteases and cellulases. For instance, Viscozyme was utilised to obtain a high-yield bilberry extract with enhanced antioxidant capacity (Syrpas et al. 2021). Alcalase was used to generate bioactive peptides from mesopelagic fish protein hydrolysates (Hayes et al. 2024). In a study on lentil proteins, the highest phenolic compound content (3.8 mg GAE/g) was achieved via sequential hydrolysis with Alcalase and Flavourzyme (Rezvankhah et al. 2021).

Additionally, studies suggest that fermentation enhances antioxidant capacity by breaking down complex or bound antioxidant compounds into more bioavailable and active forms, and by generating new bioactive molecules such as peptides and vitamins (Verni et al. 2019). These effects depend on the specific metabolic activities of the microbial strains used. Overall, these findings demonstrate that combining enzymatic pretreatment with microbial fermentation is an effective strategy for improving the biofunctional value of *C. vulgaris*. From an application perspective, such improvements expand the potential of microalgal biomass as a natural antioxidant source for food fortification, nutraceuticals, and cosmetic formulations, areas where stability and natural origin are increasingly prioritised.

### Antimicrobial activity of fermented *C. vulgaris*

Alternative antimicrobial products have gained increasing attention as a response to rising drug resistance and environmental concerns. As a result, the antimicrobial potential of microalgae as a source of sustainable and effective bioactive compounds for diverse applications has been explored (Xia et al. 2021). In fact, extracts from *C. vulgaris* exhibit antimicrobial properties (Kitada et al. 2009; Fassi Fihri et al. 2024), making this species a promising candidate for the development of functional ingredients for food and pharmaceutical use (Yuan et al. 2020). To assess this potential, the antimicrobial activity of fermentation supernatants was evaluated against five bacterial strains (Table 3). Among the tested samples, only Viscozyme-treated samples fermented with *L. plantarum* exhibited inhibition zones under the above screening conditions. For comparison, Table 3 includes enzyme-treated but not fermented (VPL and VAPL) samples, which showed no activity. Nevertheless, it should be noted that all other tested conditions (i.e., untreated but fermented samples) showed no activity.

The strongest activity was observed in the Viscozyme-treated sample (FVPL), which inhibited all strains except *P. aeruginosa*, with zones ranging from 8.6 mm (*S. aureus*) to 12.4 mm (*M. luteus*). Moderate inhibition was noted against *E. coli* (Table 3). Enzymatic hydrolysis using Alcalase did not enhance antimicrobial properties, as no inhibitory effects were detected in Alcalase-treated samples.

**Table 3:** Antimicrobial activity of enzyme-treated and fermented *C. vulgaris* by *L. plantarum* and *L. brevis*

	Inhibition zone, mm				
	*S. aureus*	*B. subtilis*	*M. luteus*	*E. coli*	*P. aeruginosa*
VPL	0.00 ± 0.00	0.00 ± 0.00	0.00 ± 0.00	0.00 ± 0.00	0.00 ± 0.00
VAPL	0.00 ± 0.00	0.00 ± 0.00	0.00 ± 0.00	0.00 ± 0.00	0.00 ± 0.00
FVPL	8.64 ± 0.01	10.37 ± 0.06	12.38 ± 0.13	9.64 ± 0.03	0.00 ± 0.00
FVAPL	7.79 ± 0.13	7.90 ± 0.05	9.54 ± 0.37	0.00 ± 0.00	0.00 ± 0.00

Results represent mean ± SD. VPL Viscozyme treated biomass unfermented; VAPL: combined treated unfermented; FVPL: Viscozyme treated and fermented by *L. plantarum*; FVAPL: combined enzyme treated and fermented by *L. plantarum*

These findings are consistent with previous work on macroalgae, in which Alcalase treatment failed to confer antimicrobial activity across the species tested (Habeebullah et al. 2020). The activity observed in Viscozyme-treated samples likely reflects efficient carbohydrate release, supporting the higher metabolic activity of *L. plantarum*. Generally, the antimicrobial effects of LAB-fermented products are linked to organic acid production, peptide or proteinaceous bacteriocins, and other small molecules (Ibrahim et al. 2021). Among these, lactic acid is notable for lowering pH and permeabilising the membranes of Gram-negative bacteria, such as E. coli, thereby enhancing the action of other antimicrobial compounds (Alakomi et al. 2000). However, fermentate activity typically cannot be attributed to a single molecule (Figueroa et al. 2024). In fact, the absence of activity in *L. brevis*-fermented samples suggests that the antimicrobial effect of *L*. *plantarum* may reflect plantaricin‑type bacteriocins, among other factors, with definitive attribution requiring neutralisation, catalase and protease treatment assays, which were outside the scope of this screening. Many *L. plantarum* strains synthesise plantaricins, bacteriocins of interest for their diverse applications, including food biopreservation, mitigation of irritable bowel syndrome symptoms, and protection against urinary tract infections (Abdulhussain Kareem and Razavi 2020). *L. plantarum* strains that produce plantaricins are valued for their gut-friendly characteristics, broad carbohydrate utilisation, pleasant sensory profile, and probiotic properties, making them highly suitable for applications in the food industry (Goel and Halami 2023).

## Conclusions

This work demonstrates that enzymatic pretreatment combined with lactic acid fermentation is an effective and eco-friendly strategy to enhance the bioactivity of *C. vulgaris* biomass. Among the tested pretreatment methods, enzymatic hydrolysis, particularly the combined use of Viscozyme and Alcalase, effectively disrupts the rigid cell wall, thereby increasing the release of fermentable sugars and soluble proteins. This enhanced nutrient availability improves substrate accessibility for LAB, thereby supporting efficient growth, promoting microbial metabolism and the production of bioactive metabolites. Fermentation further contributed to functional improvements, notably increased antioxidant capacity and screening-level inhibitory effects under non‑neutralised conditions against selected Gram-positive bacteria following Viscozyme pretreatment and *L. plantarum* fermentation. These results demonstrate the potential of integrated bioprocessing for valorising microalgal biomass into high-value biofunctional ingredients. Although enzymatic hydrolysis proved highly effective in this study, the reliance on enzymes, which may require careful handling and precise optimisation, suggests that future research could also investigate alternative or complementary pretreatment strategies to further enhance nutrient release from *C. vulgaris* biomass. In addition, future work should focus on scaling up the process, optimising microbial strain selection, and exploring metabolomic profiling to better understand the mechanisms underlying bioactivity enhancement.

## Supplementary Information

Below is the link to the electronic supplementary material.

Supplementary Material 1.

## Data Availability

All data generated or analysed during this study are included in this published article [and its supplementary information files].
