# Supplementary material for: Enhancing bioactivity of Chlorella vulgaris through enzymatic pretreatment and lactic acid fermentation
Source: Bioresour Bioprocess. 2026 Apr 6;13(1):50. doi: 10.1186/s40643-026-01050-3 (PMC13053731; doi:10.1186/s40643-026-01050-3)
Supplement: Supplementary file 1 — Supplementary Material 1. [file 40643_2026_1050_MOESM1_ESM.docx]

**Supplementary Materials**

**Enhancing bioactivity of *Chlorella vulgaris* through enzymatic pretreatment and lactic acid fermentation**

Hakki Bilgin^1^, Shahana Aboobacker^1^, Aušra Šipailienė^1^, Vaida Kitrytė-Syrpa^1^, and Michail Syrpas^1*^


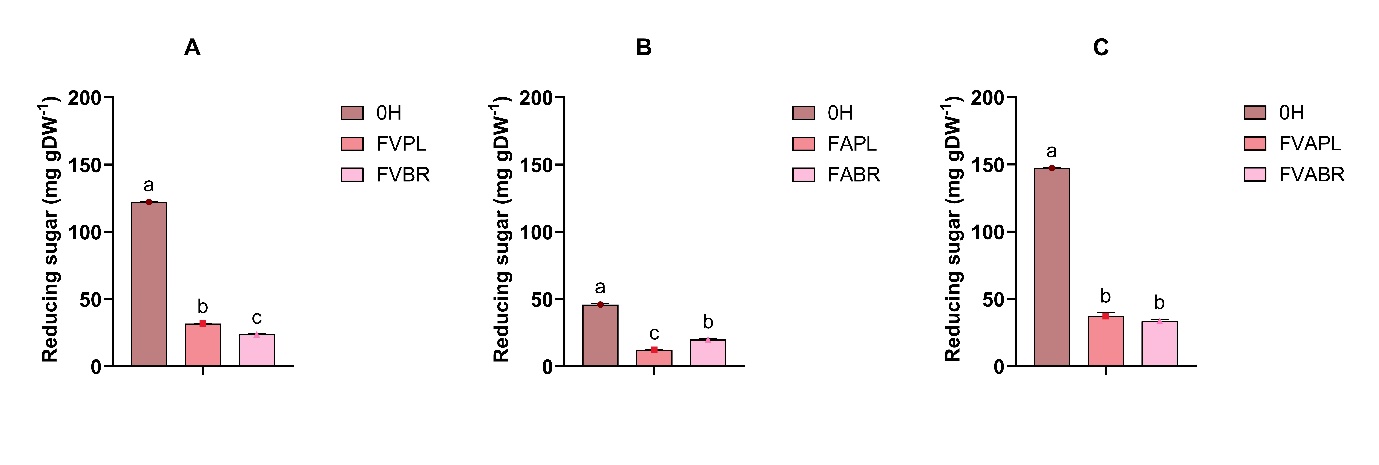


Figure S 1Reducing sugar (A-C) concentration after fermentation with L. plantarum (PL) and L. brevis (BR). (UF: unfermented; F: fermented; VC, VPL, VBR: viscozyme treated control, fermented by L. plantarum and L. brevis, respectively; AC, APL, ABR: alcalase treated control, fermented by L. plantarum and L. brevis, respectively; VAC, VAPL, VABR: combined enzyme treated control, fermented by L. plantarum and L. brevis, respectively). Bars represent means ± SD. Different letters indicate statistically significant differences (P < 0.05) (n = 3).


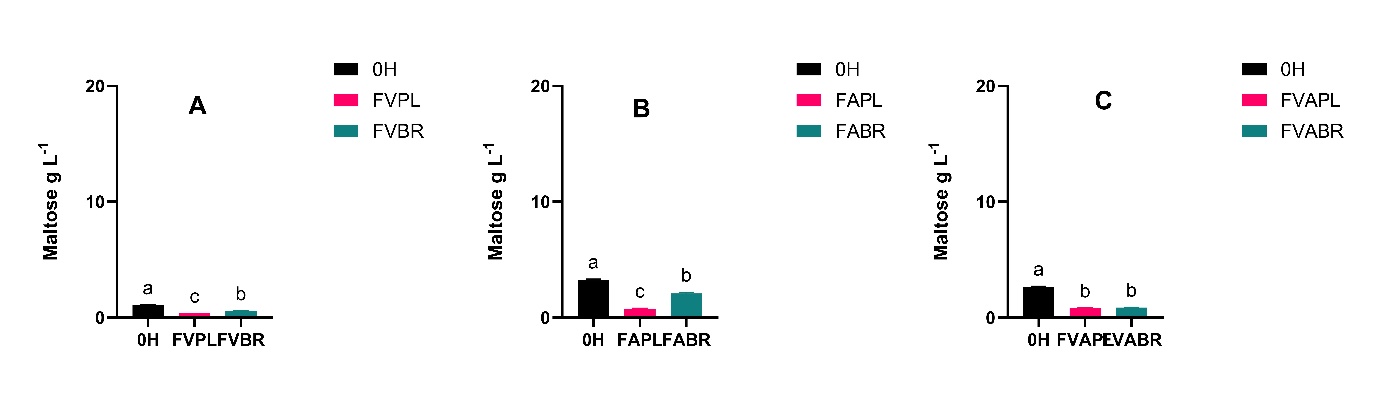


Figure S 2. Maltose concentration (A-C) after fermentation with L. plantarum (PL) and L. brevis (BR). (UF: unfermented; F: fermented; VC, VPL, VBR: viscozyme treated control, fermented by L. plantarum and L. brevis, respectively; AC, APL, ABR: alcalase treated control, fermented by L. plantarum and L. brevis, respectively; VAC, VAPL, VABR: combined enzyme treated control, fermented by L. plantarum and L. brevis, respectively). Bars represent means ± SD. Different letters indicate statistically significant differences (P < 0.05) (n = 3).


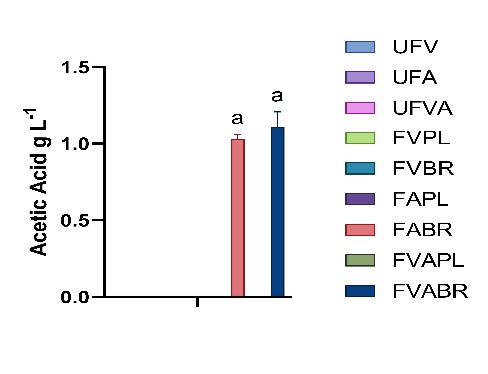


Figure S 3 Acetic acid concentration after fermentation with L. plantarum (PL) and L. brevis (BR). (UF: unfermented; F: fermented; VC, VPL, VBR: viscozyme treated control, fermented by L. plantarum and L. brevis, respectively; AC, APL, ABR: alcalase treated control, fermented by L. plantarum and L. brevis, respectively; VAC, VAPL, VABR: combined enzyme treated control, fermented by L. plantarum and L. brevis, respectively). Bars represent means ± SD. Different letters indicate statistically significant

Fig. S 4. In vitro antioxidant capacity of C. vulgaris as evaluated by the ABTS (A-C), and DPPH (D-F) assay concentration after fermentation with L. plantarum (PL) and L. brevis (BR). (UF: unfermented; F: fermented; VC, VPL, VBR: viscozyme treated control, fermented by L. plantarum and L. brevis, respectively; AC, APL, ABR: alcalase treated control, fermented by L. plantarum and L. brevis, respectively; VAC, VAPL, VABR: combined enzyme treated control, fermented by L. plantarum and L. brevis, respectively). Bars represent means ± SD. Different letters indicate statistically significant differences (P < 0.05(n = 3).
